# Quantification of the Ionic Character of Multiconfigurational Wave Functions: The *Q*_a_^t^ Diagnostic

**DOI:** 10.1021/acs.jpca.3c05559

**Published:** 2023-10-18

**Authors:** Silmar A. do Monte, Rene F. K. Spada, Rodolpho L. R. Alves, Lachlan Belcher, Ron Shepard, Hans Lischka, Felix Plasser

**Affiliations:** † Departamento de Química, CCEN, Universidade Federal da Paraíba, 58051-900 João Pessoa, Brazil; ‡ Departamento de Física, Instituto Tecnológico de Aeronáutica, 12.228-900 São José dos Campos, São Paulo, Brazil; § Chemical Sciences and Engineering Division, Argonne National Laboratory, Lemont, Illinois 60439, United States; ∥ Department of Chemistry and Biochemistry, Texas Tech University, Lubbock, Texas 79409-1061, United States; ⊥ Department of Chemistry, Loughborough University, Loughborough LE11 3TU, U.K.

## Abstract

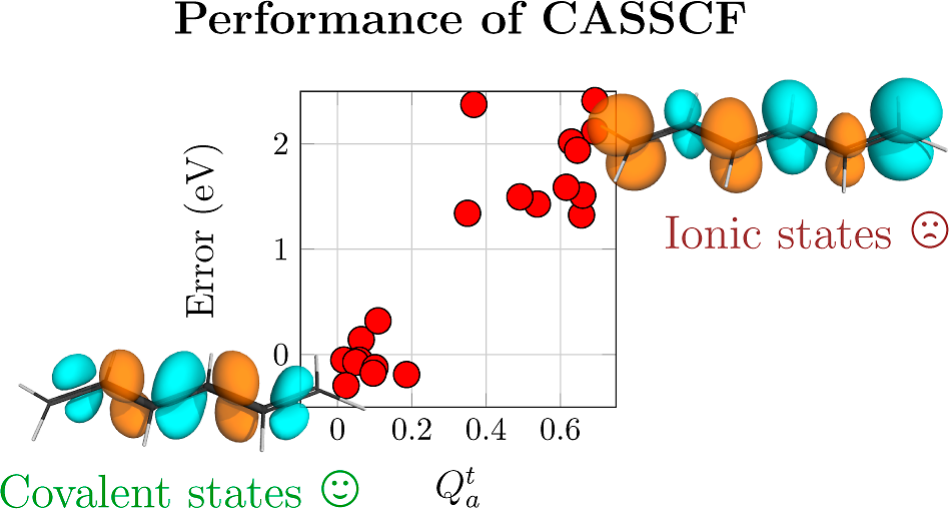

The complete active space self-consistent field (CASSCF) method is a cornerstone in modern excited-state quantum chemistry providing the starting point for most common multireference computations. However, CASSCF, when used with a minimal active space, can produce significant errors (>2 eV) even for the excitation energies of simple hydrocarbons if the states of interest possess ionic character. After illustrating this problem in some detail, we present a diagnostic for ionic character, denoted as *Q* _a_^t^, that is readily computed from the transition density. A set of 11 molecules is considered to study errors in vertical excitation energies. State-averaged CASSCF obtains a mean absolute error (MAE) of 0.87 eV for the 34 singlet states considered. We highlight a strong correlation between the obtained errors and the *Q* _a_^t^ diagnostic, illustrating its power to predict problematic cases. Conversely, using multireference configuration interaction with single and double excitations and Pople’s size extensivity correction (MR-CISD+P), excellent results are obtained with an MAE of 0.11 eV. Furthermore, correlations with the *Q* _a_^t^ diagnostic disappear. In summary, we hope that the presented diagnostic will facilitate reliable and user-friendly multireference computations on conjugated organic molecules.

## Introduction

The multiconfigurational self-consistent field (MCSCF) method and specifically complete active space SCF (CASSCF) play an important role in modern quantum chemistry by providing a stable description of molecular excited states at distorted geometries and due to their ability to access doubly excited states.^1−4^ MCSCF is either used as a method by itself, often to run dynamics,^5,6^ or as a starting point for correlated methods such as multireference configuration interaction (MRCI) or CAS perturbation theory (CASPT2).^7,8^ Despite its popularity CASSCF experiences notorious problems in describing a specific class of excited states, the so-called ionic states as identified within valence bond theory.^9−12^ The CASSCF excitation energies of ionic states are often overestimated by about 1–2 eV, when a minimal active space of valence π and π* orbitals is used. A number of strategies have been proposed to address this problem: doubling the π-active space, inclusion of σ-orbitals into the active space, and modification of the one-electron basis set.^9,11,13−15^ However, it is not readily apparent when such a more sophisticated treatment is needed. Furthermore, a subsequent correlated treatment via MRCI or CASPT2^16,17^ is generally beneficial but the problems of CASSCF often resurface also in this case.^3,18^ Aside from CASSCF, it is worth noting that the distinction between ionic and covalent states, and more specifically the *L*_a_ and *L*_b_ states of cyclic conjugated molecules have also been invoked to assess the reliability of time-dependent density functional theory (TDDFT) and wave function-based single-reference methods.^19,20^

Ionic states are ubiquitous in molecular systems. As a rule of thumb, the HOMO/LUMO transition of any conjugated hydrocarbon possesses appreciable ionic character if there is spatial overlap between HOMO and LUMO. The same holds for conjugated hydrocarbons substituted with heteroatoms unless they possess strong donor–acceptor characteristics. However, a more detailed characterization of ionic states is challenging within the standard MO picture^21−23^ and the characterization often relies on specific valence bond methods used already for generating the wave functions.^24^ These difficulties mean that researchers are blindsided by serious and unpredictable errors in CASSCF computations without any way of detecting problematic states a priori. The issue has parallels with the notorious charge transfer (CT) problem for time-dependent density functional theory, in the sense that computations can fail dramatically leading to errors in excess of 1 eV.^25^ The CT problem of TDDFT was significantly ameliorated via the development of diagnostics that illuminate CT character and highlight when TDDFT should be used with caution.^26−29^ Moreover, diagnostics and descriptors have been developed to quantify other challenging wave function properties such as multireference character,^30−33^ doubly excited character,^34^ and orbital relaxation.^35^ All of these are helping researchers choose appropriate computational methods and to benchmark the quality of their results.^36^ However, analogous readily applicable descriptors are missing in the case of ionic states. A related development is concerned with an ionicity index based on the on-top pair density.^37^ However, this has not yet been widely adopted, most probably due to the difficulty of computing the required on-top pair density.

**Figure 1:**
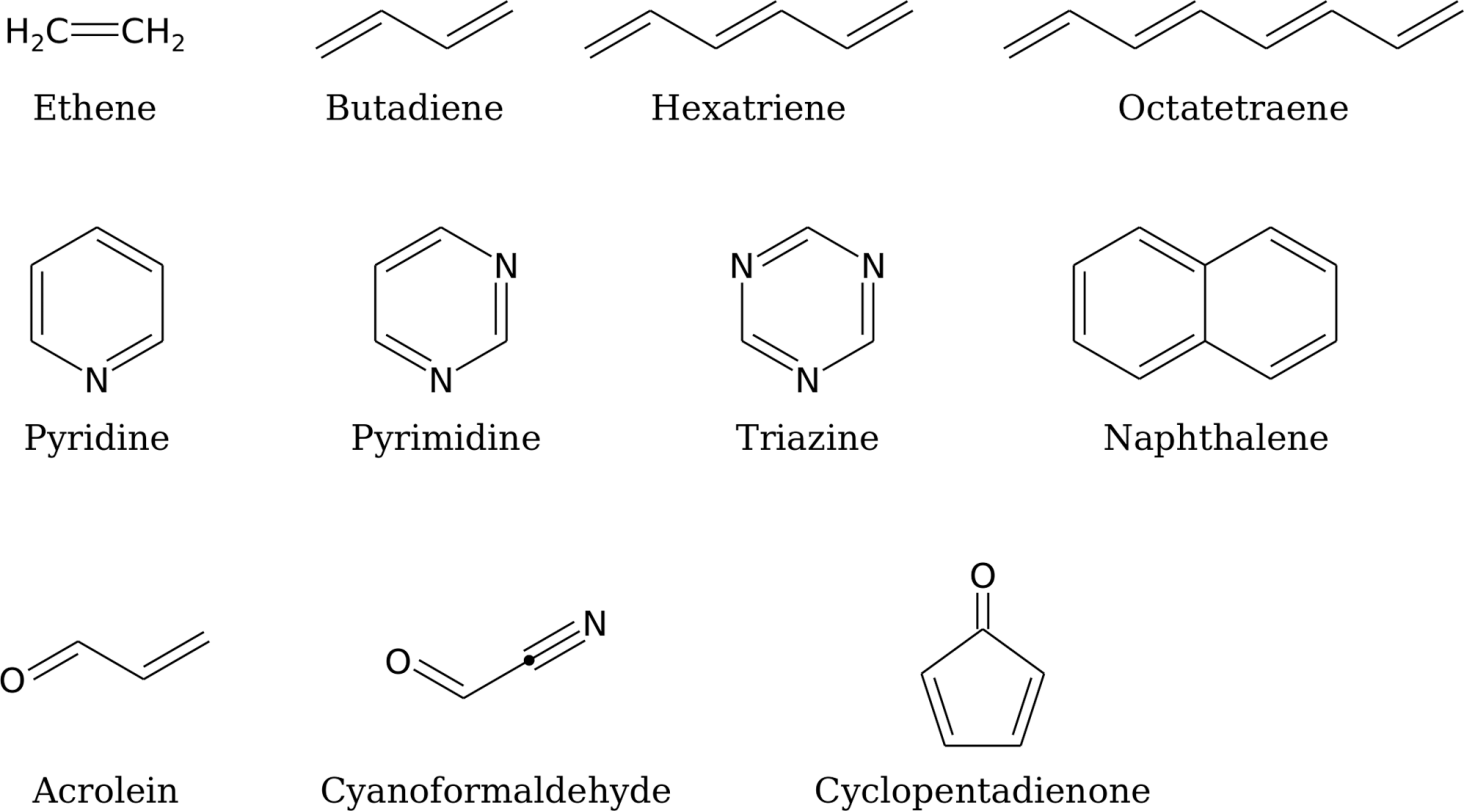
Structures of the molecules considered in this work.

Within this work, we introduce a diagnostic that is readily computed and aimed at providing information on ionic states in a wide class of molecular systems. This diagnostic is based on transition charges obtained from the one-electron transition density matrix (1TDM), which is routinely computed in quantum chemistry programs. After presenting the methods, we first highlight the underlying physics in the example of ethene and present concrete results in the case of naphthalene. Subsequently, a more general analysis is presented considering a set of 11 molecules, as presented in Figure 1. We analyze errors in vertical excitation energies considering CASSCF and various MRCI methods and correlate the results to the newly developed diagnostic.

## Methods

### Detection of Ionic States

The diagnostic developed here is based on a population analysis of the transition density to obtain the transition charges. For context it is worth noting that, within the literature, transition charges are often used to model excitonic interactions, especially in combination with electrostatic potential (ESP) fitting to obtain the TrESP charges.^38,39^ However, here, we will view them in a somewhat different light.

We compute the transition charge on atom *M* as
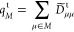
1where *D̃*_μμ_^t^ is a diagonal element of the Löwdin-orthogonalized one-electron transition density matrix (1TDM) between the state of interest and the ground state. As will be discussed below, large transition charges on individual atoms are associated with ionic states, and we will, therefore, endeavor to quantify the overall magnitude of the transition charges.

Directly summing over the transition charges is not instructive as the sum vanishes for transitions between orthogonal states^40^
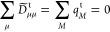
2

As an alternative, we suggest summing over the absolute values of the transition charges and define the descriptor
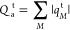
3intended to capture states where a substantial amount of transition density is located on the atoms. As a second option, we also compute the LOC_a_ descriptor where we directly take the absolute value of diagonal 1TDM elements.
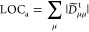
4

This is closely related to the LOC measure introduced in ref (41), only that absolute rather than squared 1TDM elements are used here. A comparison of the above definition shows that
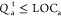
5where the difference derives from cancellations within any given atom. Both descriptors are greater than or equal to zero. Conversely, it is not trivial to deduce a maximal possible value for either descriptor; test calculations show that both descriptors may readily exceed a value of 4.0. Finally, we note that, formally speaking, *Q* _a_^t^ and LOC_a_ are both measures of charge; the values given here are in atomic units (that is multiples of the unit charge *e*).

In addition to ionic character, we will also compute the double excited character of the excited states. For this purpose, we use the squared norm of the 1TDM,^34,40,42^ computed as
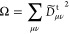
6

### Computational Details

A set of 11 molecules were investigated, as shown in Figure 1. The geometries were extracted from the quantum excited state database (QUESTDB),^43^ in which all structures were optimized for the ground state using the CC3/aug-cc-pVTZ method. Of these molecules, we analyzed vertical excitation energies of valence singlet and triplet excited states, encompassing covalent and ionic ππ* states as well as nπ* states. The precise set of excited states used is presented in Table 1. In total, we computed 36 singlet and 20 triplet excited states. Out of these, 34 singlets and 20 triplets were compared against theoretical best estimates (TBEs) from QUESTDB. The reason for this discrepancy in the number of states derives from the fact that in some cases the state-ordering in CASSCF is reversed compared to QUESTDB and, therefore, a larger number of states had to be included in order to access the ionic states of interest. States were matched against QUESTDB references based on symmetry, state character, and oscillator strengths.

**Table 1:** Details of Computations Performed: Excited States Used in the State-Averaging Procedure (Along with the Ground State) and CAS (Number of Active Electrons and Number of Active Orbitals) Used

molecule	excited states	CAS
ethene	^1^B_u_, ^3^B_u_	(2,2)
butadiene	^1^A_g_, ^1^B_u_, ^3^A_g_, ^3^B_u_	(4,4)
hexatriene	^1^A_g_, ^1^B_u_(2), ^3^A_g_, ^3^B_u_	(6,6)
octatetraene	^1^A_g_, ^1^B_u_(2), ^3^A_g_, ^3^B_u_	(8,8)
naphthalene	^1^B_3u_, ^1^B_2u_, ^3^B_3u_, ^3^B_2u_	(10,10)^a^/(8,8)^b^
pyridine	^1^B_1_, ^1^B_2_, ^1^A_2_, ^1^A_1_, ^3^A_1_	(8,7)
pyrimidine	^1^B_1_(2), ^1^A_2_(2), ^1^B_2_, ^1^A_1_, ^3^A_1_	(10,8)
triazine	^1^A_1_, ^1^B_2_, ^1^A_2_, ^1^A_1_, ^3^A_1_	(12,9)
acrolein	^1^A′(2), ^1^A″(2), ^3^A′, ^3^A″	(6,5)
cyanoformaldehyde	^1^A″(2), ^3^A′, ^3^A″	(10,8)
cyclopentadienone	^1^A_1_(2), ^1^B_1_, ^1^B_2_, ^1^A_2_, ^3^A_1_, ^3^B_1_, ^3^B_2_, ^3^A_2_	(8,7)

a For SA-CASSCF and MR-CIS.

b For MR-CISD [using SA-CASSCF(10,10) orbitals].

The states mentioned have been studied at the state-averaged complete active space SCF (SA-CASSCF), multireference configuration interaction with singles (MR-CIS), and multireference configuration interaction with singles and doubles (MR-CISD) levels. At the SA-CASSCF level, the aforementioned states (along with the ground state) have been averaged with equal weights, and the optimized molecular orbitals were then used for the subsequent MR-CIS and MR-CISD calculations. SA-CASSCF computations were performed with a standard valence CAS comprising all n, π, and π* orbitals. The same space was used as a reference for the MR-CIS and MR-CISD computations. The only exception to this was naphthalene, where the reference space for MR-CISD comprises only eight π orbitals (and eight electrons) to avoid excessive computational cost. All 1s core orbitals were frozen in the MR-CI computations.

Generalized interactive space restrictions have been used at the MR-CISD level.^44^ On the other hand, at the MR-CIS level such a restriction was not applied and all symmetries were allowed to generate the reference CSFs. Where specified, the Davidson correction^45^ extended to the multireference case^46^ has been used to take the size-extensivity error into account. The correction in its original form is named +DV1, to differentiate from its two variations also used in this work, +DV2^47,48^ and +DV3.^49,50^ Another extensivity correction, due to Pople (+P),^51^ also extended to the multireference case,^52^ has been used, as well. The SA-CASSCF, MR-CIS and MR-CISD calculations have been done with the Columbus 7.2 program system^53−56^ using integrals from the Dalton program.^57^ All calculations reported used the aug-cc-pVDZ basis set.^58^

Based on the computed excited states, we performed a 1TDM analysis using the TheoDORE 3.1.1 program package^59^ to obtain the *Q* _a_^t^, LOC_a_ and Ω indices described above (denoted QTa, LOCa, and Om in the TheoDORE output). These indices, computed with Columbus and TheoDORE are used in the bulk of this work. Where specified, we also used OpenMolcas,^60^ allowing us to compute singlet–triplet 1TDMs at the CASSCF level, and Q-Chem 6.1 for ADC(3) computations.^61,62^ In these cases, the analysis proceeded via the wave function analysis library libwfa.^63,64^*Q* _a_^t^ and LOC_a_ descriptors were generally computed by using an underlying Löwdin population analysis scheme. Selected results using Mulliken partitioning are presented in the Supporting Information (Table S1).

## Results and Discussion

### Overview of Ionic States

**Figure 2:**
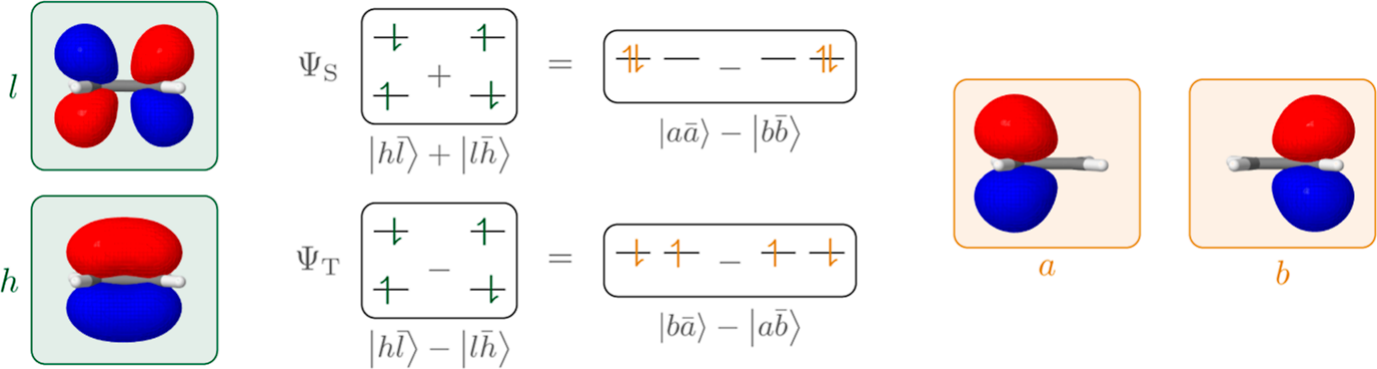
Example showing the covalent triplet and ionic singlet states of ethene using delocalized canonical MOs (left) and localized MOs (right).

It has been long recognized that the ππ* excited states of conjugated hydrocarbons can be classified into ionic and covalent states within valence-bond theory.^21−23,65−67^ Within a standard MO picture, this difference is difficult to appreciate. Therefore, we want to show a basic example of this differentiation in Figure 2, whereas a more detailed discussion is given in ref (68). Figure 2 represents the HOMO/LUMO transition in ethene considering both singlet and triplet multiplicity. The singlet is written as a combination of two Slater determinants
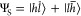
7where *h* and *l* refer to HOMO and LUMO and the bar marks β-spin. The triplet is written analogously but with a minus sign
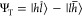
8

Expressed within localized orbitals *a* and *b*, those expressions read^22,68^
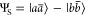
9
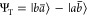
10

Crucially, the singlet in eq 9 is composed of configurations where both electrons are simultaneously on either *a* or *b*, whereas the two electrons are on opposite sites for the triplets. Hence, the singlet is classified as ionic (or zwitterionic) and the triplet as covalent (or biradical) within the valence-bond language. Importantly, neither of the states shows any permanent charge transfer. The difference between the states is a static correlation effect derived from the spin-coupling of the two determinants involved. A similar differentiation is possible for any alternant hydrocarbon due to the symmetry properties of the HOMO and LUMO.^68^ For excited states involving other orbitals than the HOMO and LUMO, Pariser’s “±” nomenclature is often applied where “+” states are interpreted as ionic and “–” states as covalent.^21,41,65,66^

A similar differentiation between ionic and biradical states is also possible for heteroaromatic molecules if the introduction of the heteroatom is seen as only a small perturbation preserving the approximate symmetry of the pure hydrocarbon structure. Most commonly, the labels *L*_a_ and *L*_b_, as introduced initially by Platt,^69^ are used to describe the first ionic and covalent state, respectively.^68^

From a computational point of view the crucial realization is that ionic states require enhanced σ-correlation in their description.^11,12,14^ This explains why CASSCF, when only including n/π/π* orbitals, can drastically fail in the description of ionic states. In addition, the orbitals produced for ionic states by CASSCF can be too diffuse meaning that any subsequent correlation treatment has a suboptimal starting point.^12^

As an alternative to the above discussion, it is also possible to appreciate the problem described within the delocalized MO picture. In this context, it is worth realizing that the singlet excited state is destabilized by the self-repulsion of the transition density^41,70^ (often approximated as the HOMO/LUMO exchange integral^71,72^), whereas the triplet is not. Furthermore, singlets mix σ-contributions into the transition density to lower this repulsion term and this provides a graphic signature of the σ-contributions affecting ionic states.^41,70^ An example of this is shown below.

### Example of Naphthalene

To start the discussion on concrete computations, we present some results on naphthalene serving as a paradigmatic alternating hydrocarbon, allowing us to illustrate the main ideas behind our strategy. The excitation energies of the first two singlet and triplet states using various quantum chemical methods are presented in Table 2. Comparing SA-CASSCF in the first column and the theoretical best estimate (TBE) from ref (43) in the last column, we find that the first three states are described fairly well whereas the energy of the last state, ^1^B_2u_^+^ (^1^L_a_), is dramatically overestimated, lying at 6.41 eV, which is 1.5 eV higher than the TBE. At the MR-CISD level we find that the first triplet ^3^B_2u_^+^ (^3^L_a_) also lies within 0.2 eV of the TBE. The singlet and triplet B_3u_ states, incidentally are described somewhat worse, but still lying within 0.5 eV of the reference. MR-CISD finally provides a significant improvement to the description of the second singlet, ^1^B_2u_^+^ (^1^L_a_), which is reduced to 5.67 eV but still about 0.7 eV too high. If an appropriate extensivity correction is used for MRCI, in this case the Pople correction (+P),^51^ then the results are greatly improved and all excitation energies lie within 0.1 eV of the TBE values. For later reference, we show the ADC(3) results, which also lie close to the TBE values but show a somewhat larger spread than MRCISD+P.

**Table 2:** Vertical Excitation Energies for Naphthalene Computed for the Lowest Lying B_2u_ and B_3u_ States of Singlet and Triplet Multiplicity Using Various Computational Levels in Connection with the aug-cc-pVDZ Basis Set

state	SA-CASSCF	MR-CISD	MR-CISD+P	ADC(3)	TBE^a^
^3^B_2u_^+^ (^3^L_a_)	3.03	3.32	3.28	2.95	3.17
^3^B_3u_^+^ (^3^B_b_)	4.28	4.59	4.25	3.94	4.16
^1^B_3u_^–^ (^1^L_b_)	4.22	4.80	4.35	4.21	4.27
^1^B_2u_^+^ (^1^L_a_)	6.41	5.67	4.99	4.82	4.90

a Theoretical best estimate, from ref (43), aug-cc-pVTZ basis set.

**Figure 3:**
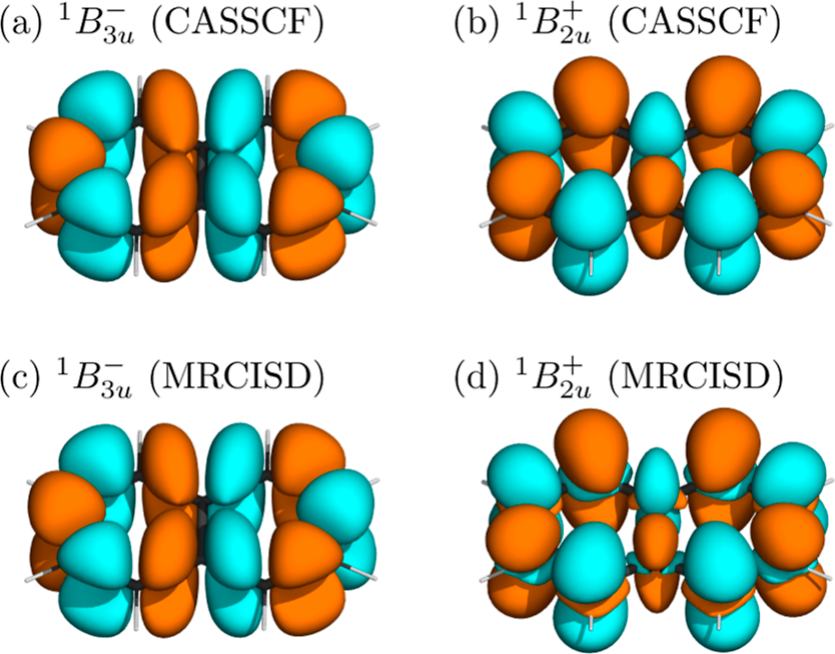
Transition densities between the ground state and the lowest two singlet states of naphthalene computed at SA-CASSCF (a,b) and MRCISD (c,d) levels of theory (isovalue: 0.001 au).

Notably, all four of the states discussed are ππ* states delocalized over the whole naphthalene molecule. It is therefore not immediately apparent what sets apart the ^1^B_2u_^+^ state in terms of causing challenges in the SA-CASSCF description. However, as alluded to above, the problem with this state is its ionic nature. The difference between covalent and ionic states can be appreciated in terms of the transition densities computed with respect to the ground state.^19,41,68,73^ The transition densities of biradical states are centered around the bonds, whereas they are localized on the atoms for ionic states. This is illustrated for naphthalene in Figure 3 highlighting the example of the biradical ^1^B_3u_^–^ and ionic ^1^B_2u_^+^ states in panels (a) and (b), respectively. We will use this information to devise a numerical diagnostic below. However, before moving on, we want to describe a curious property of the MRCISD transition densities (Figure 3c,d). We find that the transition density for ^1^B_3u_^–^ is almost unaltered when compared to that for SA-CASSCF. Strikingly, this is not the case for the ^1^B_2u_^+^ state, where new contributions in the σ-system appear. The appearance of these σ-contributions is a general phenomenon for ionic ππ* states and was also seen in TDDFT and ADC computations.^41,70,74^ A detailed interpretation of their occurrence and a quantitative analysis of the excitation energy components involved has been given in refs (41),^70^, and here, only a short explanation will be given. First, it is noteworthy that the energy of singlet excited states is increased by a term that is proportional to the transition density self-repulsion. Second, this repulsive term can be compensated by σσ* excitations yielding opposing terms in the transition density. More generally, these contributions are a signature of the σ-correlation associated with ionic states. Crucially, for symmetry reasons, it is fundamentally impossible to capture this effect in an π-only CAS computation.

The pictorial representations of the transition densities provide a path forward as to how to differentiate biradical and ionic states, considering that these are located either on the bonds or on the atoms, respectively. Based on this knowledge, we now attempt to devise a numerical diagnostic that allows us to determine a state’s character without the need of a visual inspection of the transition density. Here, we suggest two related diagnostics for this purpose, the sum over absolute transition charges *Q* _a_^t^ [eq 3] and the sum over absolute diagonal density matrix elements LOC_a_ [eq 4]. Viewing Figure 3a, it can be appreciated that for any given atom, transition density contributions from either side will approximately cancel out, leaving a net zero transition charge on this atom. Conversely, the ionic state in Figure 3b has clearly nonvanishing transition charges on each atom. The rationale behind LOC_a_ is similar in only that it is a more fine-grained quantity adding up contributions from each basis function individually. The *Q* _a_^t^ and LOC_a_ values, computed at the SA-CASSCF, MRCISD, and ADC(3) levels of theory are presented in Table 3. Importantly, there is a clear distinction between the states classified as “+” and “–”. The ionic “+” states have *Q* _a_^t^/LOC_a_ values above 0.3/1.0 for all methods considered. By contrast, *Q* _a_^t^ and LOC_a_ are consistently below 0.05 and 0.2, respectively, for the covalent ^1^B_3u_^–^ state. This suggests that *Q* _a_^t^ and LOC_a_ are indeed suitable descriptors for ionic character, and we will evaluate the generality of this statement below.

**Table 3:** Diagnostics for Ionic Character (*Q* _a_^t^ and LOC_a_) for the Naphthalene Molecule are Computed at the SA-CASSCF, MR-CISD, and ADC(3) Levels of Theory Using an Underlying Löwdin-Style Population Analysis

state	SA-CASSCF	MR-CISD	ADC(3)	SA-CASSCF	MR-CISD	ADC(3)
	*Q* _a_^t^	*Q* _a_^t^	*Q* _a_^t^	LOC_a_	LOC_a_	LOC_a_
^3^B_2u_^+^	1.935^a^		1.406	3.133^a^		2.721
^3^B_3u_^+^	1.127^a^		1.020	1.372^a^		1.351
^1^B_3u_^–^	0.017	0.024	0.022	0.104	0.122	0.188
^1^B_2u_^+^	0.660	0.581	0.394	1.285	1.614	2.016

a Computed using OpenMolcas.

Before continuing, we want to make a brief comment about the triplet states. Table 3 shows that the *Q* _a_^t^ values for the triplets are considerably higher than the values for the singlets. First, it is worth realizing that the lowest B_3u_ triplet is a “+” state, whereas the B_3u_ singlet is a “–” state^41,68^ and this is the reason for its strongly altered *Q* _a_^t^ value. The different character of this state is also seen in the different form of its transition density (see Figure S1). The singlet and triplet B_2u_ states are both strongly dominated by the HOMO/LUMO transition and are analogous to the *L*_a_ and “+” states. Nonetheless, the presented analysis shows that there is a quite substantial difference in the wave functions of these states other than simply the change in spin. The lower *Q* _a_^t^ values of the singlets reflect the drive of the singlets to avoid exchange repulsion. The differences between the singlet and triplet B_2u_ states are for example also represented by slightly altered transition densities (see Figure S1) and different natural orbital (NO) occupation patterns; the formal HOMO and LUMO possess NO occupations of 1.08/0.95 for the singlet and 1.18/0.83 for the triplet. However, *Q* _a_^t^ and LOC_a_ are arguably more sensitive measures for highlighting such changes in the wave functions. A more detailed related analysis, highlighting how the various singlet and triplet states differ in push–pull systems, is presented in ref (75).

We also want to highlight that it is an attractive property of the presented analysis that the trends are consistent across the computational methods. This means that single-reference jobs, such as TDDFT or ADC can be used to gauge whether ionic states are present for a given molecule in the desired energy range.

Finally, we have recomputed the data from Table 3 using Mulliken-style (rather than Löwdin-style) population analysis (Table S1). Using a Mulliken-style analysis, we obtain similar trends with *Q* _a_^t^ and LOC_a_ both being higher for ionic than for covalent states. However, there is some variation between the absolute values obtained and it is particularly noteworthy that the Mulliken values are generally higher than the Löwdin ones. Similarly, we would expect that *Q* _a_^t^ values based on another population analysis approach (e.g., TrESP charges or projection into a minimal basis set) would yield altered results. We do not believe that this discrepancy has any effect on the conclusions of this paper but we want to stress that particular care is required when comparing data between different computational methods, quantum chemistry codes and, in particular, basis sets to ensure that all data are consistent.

### Data Set

**Figure 4:**
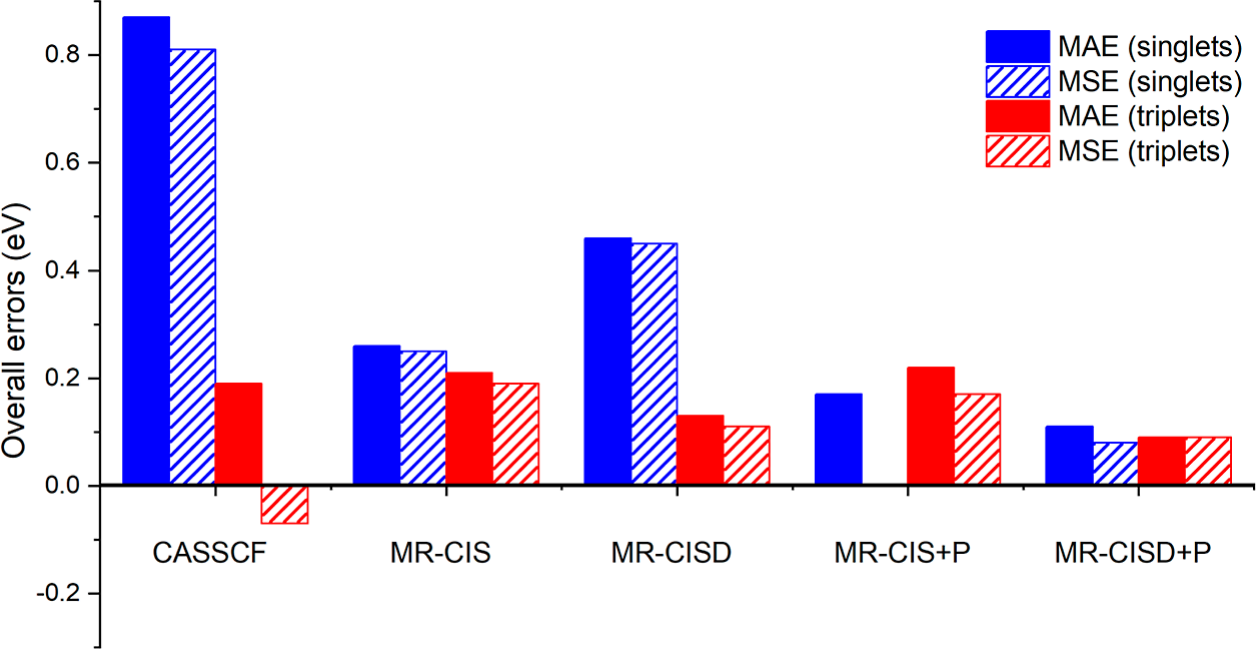
Overall errors of vertical excitation energies for SA-CASSCF and various MR-CI variants (using the aug-cc-pVDZ basis set) computed for the 11 molecules considered in this work. Results are reported as mean absolute and signed errors (MAE, MSE) determined separately for singlet and triplet states.

To evaluate the generality of the above statements, we investigate a set of 11 molecules (Figure 1), which form a subset of the QUESTDB database of excitation energies.^43^ The molecules chosen form a varied set comprising linear and cyclic conjugated hydrocarbons as well as molecules substituted with nitrogen and oxygen. The energies of 34 singlet states and 20 triplet states are evaluated and compared to QUESTDB reference values. The overall errors for SA-CASSCF and various MR-CI variants are listed in Figure 4. A mean absolute error (MAE) of 0.87 eV is obtained for SA-CASSCF for singlet states highlighting the challenges of this method in obtaining accurate excitation energies. The largest errors are well above 2 eV (see below). The strongly positive mean signed error (MSE) in Figure 4 in the case of SA-CASSCF singlets highlights that the excitation energies are generally overestimated. The description of the triplets is significantly better giving MAE/MSE of only 0.19/–0.07 eV. Continuing with MR-CIS, we find that the errors for singlets are significantly reduced giving MAE/MSE of 0.27/0.25 eV, whereas the MAE of the triplets is largely unaltered. Moving to the computationally significantly more expensive MR-CISD level, we find that the description of the singlets actually deteriorates producing an MAE/MSE of 0.46/0.45 eV. Conversely, the triplets are improved with a very low MAE/MSE of 0.13/0.11 eV. In summary, the methods discussed so far, all describe triplets well, whereas singlets, and in particular the ionic states, are quite critical.

The challenges in describing ionic states are long known and extensivity corrections to MR-CI have proven as an effective strategy to mitigate them.^3,16^ A number of extensivity corrections have been applied, and all results are shown in Figure S2. We find that extensivity corrections greatly improve the MR-CISD results while having a somewhat smaller impact on MR-CIS. Considering MR-CISD, we find that there is a notable dependence on the precise scheme used, with the more sophisticated schemes (+DV3 and +P) generally offering better results than the older schemes (+DV1 and +DV2). For brevity, only the +P results are shown in Figure 4. MR-CIS+P offers an improvement for singlets in terms of its MAE (reducing it to 0.17 eV), whereas the triplets are almost unaltered (MAE of 0.22 eV). MR-CISD+P, finally, provides reliable results across the board, with MAEs of 0.11 and 0.09 eV for singlets and triplets, respectively.

At this point, we believe it is worth highlighting the excellent performance of MR-CISD+P. With an overall MAE of 0.10 eV over our data set, it performs notably better than commonly used excited-state methods, such as CC2, ADC(2), and even ADC(3) all with errors above 0.15 eV (as reported in ref (43), evaluated over the whole QUESTDB data set). These excellent results are obtained without even fully taking care of basis-set incompleteness; i.e., the results presented are at the aug-cc-pVDZ level, whereas QUESTDB uses aug-cc-pVTZ. This certainly points to MR-CISD+P being an accurate method that is robust even for challenging cases, such as double excitations and distorted geometries. Conversely, we note that MR calculations always possess an additional level of difficulty, as the results obtained depend on the active space as well as on the states in the averaging procedure (see details for both in Table 1). Moreover, we note that unlike the underlying MR-CISD method, MR-CISD+P is no longer fully variational. This means that the computation of properties and energy gradients is significantly more involved and is not routinely available. Nonetheless, we believe that the obtained results are encouraging and underline the lasting importance of extensivity corrected MR-CI.

**Figure 5:**
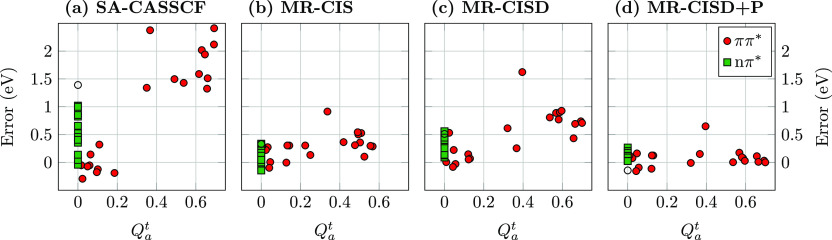
Errors of computed vertical singlet excitation energies plotted against the *Q* _a_^t^ diagnostic measuring ionic character using (a) SA-CASSCF, (b) MR-CIS, (c) MR-CISD, and (d) MR-CISD + P, all using the aug-cc-pVDZ basis set. States are grouped according to type: ππ* (circles) and nπ* (squares) states; the out-of-plane ππ* state of cyanoformaldehyde is shown as an empty circle.

Reviewing Figure 4, we now want to revisit the challenges faced by CASSCF in describing ionic states. In the following, we will discuss singlet excited states, noting that triplets are less troublesome. Figure 5 presents the errors in excitation energies plotted against the newly developed *Q* _a_^t^ diagnostic. States are grouped in terms of their character as ππ* states and nπ* states. Starting with the ππ* states at the SA-CASSCF level (filled red circles in Figure 5a), we find that the diagnostic performs just as desired. The states are divided into two groups featuring *Q* _a_^t^ values below 0.2 and above 0.3, respectively. We find that all states in the first group, i.e., the covalent ππ* states, lie within 0.5 eV of the reference. By contrast, all states of the second group, the ionic ππ* states are overestimated by at least 1.3 eV with errors going well beyond 2 eV (for the ionic ππ* states of butadiene, hexatriene, acrolein, and cyclopentadienone). The only partial exception to this rule is the out-of-plane ππ* state in cyanoformaldehyde, shown as an empty circle to the left in Figure 5a, which has a *Q* _a_^t^ value of zero for symmetry reasons. Nonetheless, this state possesses the physical characteristics of an ionic state and is overestimated by 1.4 eV similar to the lowest ππ* state of ethene. Moving on to the nπ* states (green squares), we find errors ranging between −0.2 and 1.0 eV. These errors are generally larger than those for the covalent ππ* states but smaller than those for the ionic ππ* states. We find that, for symmetry reasons, the *Q* _a_^t^ values of nπ* states in planar molecules are exactly zero and are therefore not suited for any further discrimination.

Proceeding to MR-CIS (Figure 5b), we find that all errors are substantially reduced. All states except for one lie within 0.55 eV of the reference. The exception is the 3^1^A_1_ (ππ*) state of cyclopentadienone, which is overestimated by 0.91 eV. This state yields the largest error in all four panels of Figure 5, generally located around *Q* _a_^t^ = 0.4. We closely checked the results and could not find any obvious inconsistency in this computation. We note however its unique characteristic of having partial ionic character (*Q* _a_^t^ ≈ 0.4) and partial doubly excited character (Ω ≈ 0.6, see below), which possibly induces the observed problems. Further variations in the active space or state-averaging might improve the description of this state, but we chose to leave the state in the data set as is, as a real-life example of challenges occurring. Moving back to the overall shape of Figure 5b, we find that there is no discernible influence of the *Q* _a_^t^ diagnostic and the errors are distributed fairly evenly along the whole range for ππ* and nπ* states. It is also noteworthy that the observed *Q* _a_^t^ values are generally lowered when compared to SA-CASSCF, as can be appreciated by the fact that no *Q* _a_^t^ values above 0.6 are observed at this level. This is a signature of the σ-polarization highlighted in Figure 3d, which yields an overall lowering of the transition charges on the individual atoms.

Whereas the errors associated with the ionic states were reduced by MR-CIS, they increase again somewhat when MR-CISD is used. Comparing Figure 5c,b we find that the left side of the plot, representing the covalent and nπ* states is largely unaltered. By contrast, the energies of the ionic states go up again. This can be understood by realizing that ionic states require single σσ* excitations,^70^ which are specifically provided by MR-CIS, whereas MR-CISD also has a pronounced effect on the ground states. Nonetheless, a notable improvement with respect to SA-CASSCF is observed, as no state (except for the 3^1^A_1_ state of cyclopentadienone) is overestimated by more than 1 eV. Finally, a significant improvement is observed once the Pople correction is used (MR-CISD+P, Figure 5d) providing excellent results with almost all states within 0.2 eV of the reference.

**Figure 6:**
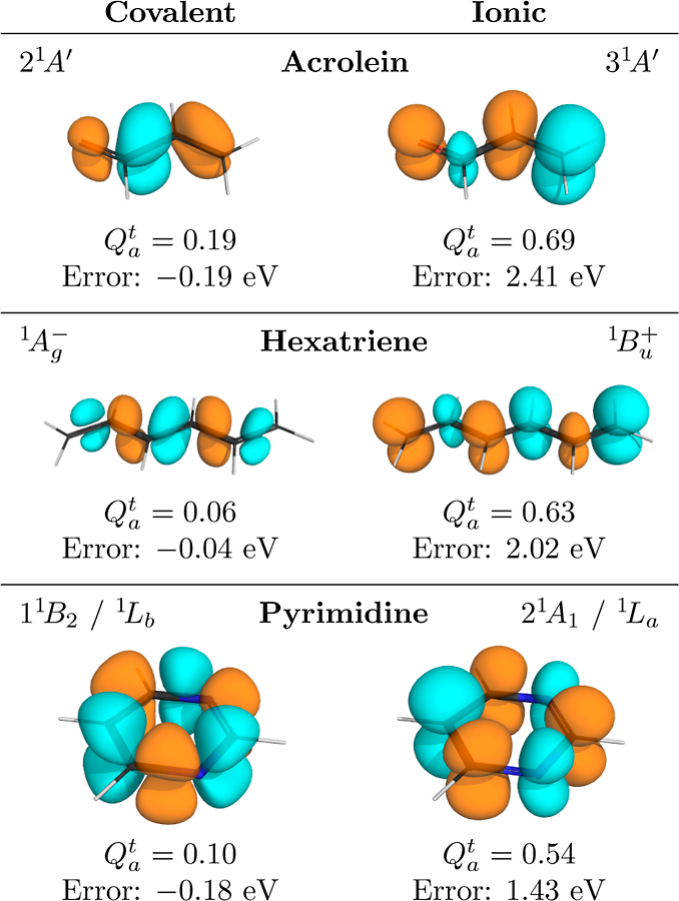
Comparison of the transition densities of the covalent and ionic ππ* states for three selected molecules computed at the SA-CASSCF/aug-cc-pVDZ level. *Q* _a_^t^ descriptors and errors of vertical excitation energies are given below each plot.

Figure 6 illustrates the relation among the transition density, the *Q* _a_^t^ descriptor, and the error in the vertical excitation energy for three selected molecules. Covalent states are shown on the left, ionic states on the right. The difference between these two types of states is clearly apparent as the transition densities are localized on the bonds in the first case and on the atoms in the second case. As a consequence, clearly different *Q* _a_^t^ descriptors are obtained, being below 0.2 for the covalent states and above 0.5 for the ionic states. The *Q* _a_^t^ descriptors correlate with the errors, which are significantly lower for the covalent states than for the ionic states.

For comparison, we have also performed the same kind of analysis for the LOC_a_ diagnostic as defined in eq 4, see Figure S3. Generally speaking, LOC_a_ offers a similar possibility for discrimination between covalent and ionic states as *Q* _a_^t^. However, the interpretation of the results is somewhat more challenging as the range of values changes; additional correlation at the MR-CI level provides for additional 1TDM elements, creating larger overall LOC_a_ values. For these reasons, we suggest using *Q* _a_^t^ as the first choice for addressing ionic states.

**Figure 7:**
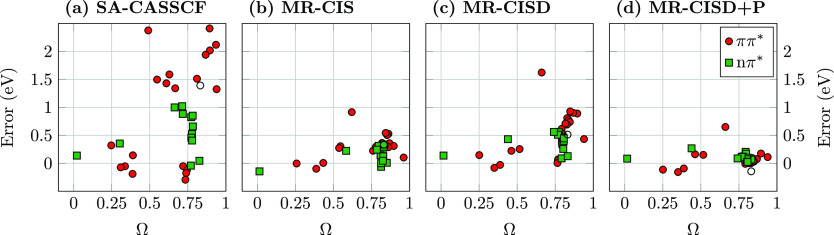
Errors of computed vertical singlet excitation energies plotted against the Ω descriptor measuring single excitation character using (a) SA-CASSCF, (b) MR-CIS, (c) MR-CISD, and (d) MR-CISD + P, all using the aug-cc-pVDZ basis set. States are grouped according to type: ππ* (circles) and nπ* (squares) states; the out-of-plane ππ* state of cyanoformaldehyde is shown as an empty circle.

Finally, we were interested in the performance of the different methods with respect to the double excitation character of the excited state. For this purpose, we use the Ω descriptor,^34,40^ as defined in eq 6. Values of Ω above 0.75 indicate a predominantly singly excited state, whereas lower values indicate admixture of doubly excited character. States with Ω below 0.5 can be considered predominantly doubly (or higher) excited. The correlation of Ω with respect to the error obtained is presented in Figure 7. Starting with the SA-CASSCF results (Figure 7a), we find that states with predominant double excitation character (Ω < 0.5) are described well, the exception being again the pathological 3^1^A_1_ (ππ*) state of cyclopentadienone. Otherwise, all errors above 0.5 eV are located on the right (single-excited) side of the plot. Moving to Figure 7b–d, we find that the different MR-CI variants improve the description of the singly excited states without affecting the doubly excited states too much. Without going into too much further detail, we want to conclude that already SA-CASSCF provides a reasonably good description of the doubly excited states. Despite its failure for singly excited ionic states, which is the focus of this review, SA-CASSCF provides an effective approach toward doubly excited states. More generally, we can envisage a prescreening procedure for the states of a newly studied molecule. On the one hand, we can screen for ionic states using *Q* _a_^t^ computed with a single reference method. On the other hand, we can screen for doubly excited states using Ω at the SA-CASSCF level. Both pieces of information combined should provide a good indication of any challenging states present.

## Conclusions and Outlook

It was the purpose of this work to introduce a diagnostic that measures the ionic character of excited states. We were particularly interested in how the ionic character affects the performance of CASSCF and other multireference methods. The diagnostic proposed here, *Q* _a_^t^, is based on a Löwdin population analysis of the transition density. *Q* _a_^t^ can be readily computed in different quantum chemistry setups, requiring negligible computational effort. The implementation presented herein is available for computations with a variety of quantum chemistry codes and methods.

We started by outlining the underlying wave function properties of covalent and ionic character. In particular, we highlighted how these can be differentiated based on their transition densities, which are located on the bonds or atoms, respectively. We showed the connection from this pictorial representation to the transition charges and, ultimately, the *Q* _a_^t^ diagnostic.

To test the performance of the new diagnostic, we studied a test set of 11 molecules and computed in total 34/20 of its singlet/triplet excited states. An overall error analysis highlighted the challenges of SA-CASSCF, when based on a minimal π/n/π* active space, in yielding accurate vertical excitation energies, in particular, of singlet states. This situation was improved when MR-CIS was used but deteriorated somehow at the MR-CISD level. Finally, when an extensivity correction was used (MR-CISD+P) excellent results were obtained, with a mean absolute error of 0.1 eV. We note, however, that there is some dependency of the correction scheme used and the best results were obtained with the Davidson-Silver (+DV3) and Pople (+P) corrections, whereas other schemes yielded somewhat enhanced errors.

In a next step, we investigated the correlation between the new *Q* _a_^t^ diagnostic and errors at the different levels of theory. A strong correlation between *Q* _a_^t^ and the observed error was observed for SA-CASSCF in the case of ππ* states. ππ* states with a value of *Q* _a_^t^ below 0.2 can be considered safe, whereas all states with *Q* _a_^t^ above 0.3 were strongly overestimated in our data set (between 1.3 and 2.4 eV). For symmetry reasons, all nπ* states had *Q* _a_^t^ values of exactly zero, and no further discrimination could be made. We briefly investigated the influence of doubly excited character in our data set. It was shown that the states with predominant doubly excited character were described well; all problematic states were singly excited ionic states.

For the practical application of the new diagnostic, we envisage two use cases. First, if a state computed with CASSCF possesses a *Q* _a_^t^ value above 0.3; then, it can be immediately deduced that its energy is most probably overestimated meaning that a more sophisticated treatment of this state is needed, e.g., through enlarging the active space or adding external electron correlation. A somewhat more challenging case is present when the state ordering of covalent and ionic states is altered, which may lead to the situation that the ionic state of interest is not even included in the state averaging process. In this case, the CASSCF computation would not produce a raised *Q* _a_^t^ value since it obtains a covalent state. However, this problem could be avoided by prescreening for ionic states using TDDFT or other single-reference methods to see if and how many ionic states are present in the desired energy range. There is a complementarity between different methods where ionic states are often well-described with TDDFT whereas CASSCF is needed in other cases, such as for doubly excited states and distorted geometries.

In the long run, we hope that a deeper understanding of the wave functions of ionic states will not only allow diagnosing problems but also lead to the development of more targeted quantum chemistry approaches that provide improved excitation energies. Current work by us is concerned with the development of an MCSCF variant with a scaled exchange repulsion term. Preliminary results suggest that such an approach can indeed improve the energies of ionic states while leaving the other states largely unaltered.

## Data Availability

The data underlying this study are openly available in Loughborough University’s data repository at DOI: 10.17028/rd.lboro.23941968. The data provided consist of molecular geometries as well as input and output files from Columbus and TheoDORE for CASSCF, MR-CIS and MR-CISD computations.

## Supporting Information Available

Further analysis of naphthalene results and further analysis of errors in vertical excitation energies (PDF)
