# Supplementary material for: Quantification of the Ionic Character of Multiconfigurational Wave Functions: The Qat Diagnostic
Source: J Phys Chem A. 2023 Oct 18;127(46):9842–52. doi: 10.1021/acs.jpca.3c05559 (PMC10683019; doi:10.1021/acs.jpca.3c05559)
Supplement: Supplementary file 1 — jp3c05559_si_001.pdf [file jp3c05559_si_001.pdf]

Supporting Information for Quantification of the Ionic Character of Multiconfigurational Wave Functions: the Q_a^t Diagnostic

Silmar A. do Monte,[†] Rene F. K. Spada,[‡] Rodolpho L. R. Alves,[†] Lachlan Belcher,[‡] Ron Shepard,[¶] Hans Lischka,[*,§] and Felix Plasser[*,‖]

†*Departamento de Química, CCEN, Universidade Federal da Paraíba, 58059-900, João Pessoa, Brazil*

‡*Departamento de Física, Instituto Tecnológico de Aeronáutica, São José dos Campos, 12.228-900, São Paulo, Brazil*

¶*Argonne National Laboratory, Chemical Sciences and Engineering Division, Lemont, IL, 60439, USA*

§*Department of Chemistry and Biochemistry, Texas Tech University, Lubbock, TX 79409-1061, USA*

‖*Department of Chemistry, Loughborough University, Loughborough, LE11 3TU, United Kingdom*

E-mail: Hans.Lischka@ttu.edu; f.plasser@lboro.ac.uk

Phone: +44 1509 226946

Table S1: Diagnostics for ionic character (Q_a^t and LOC_a) of naphthalene molecule computed at the CASSCF, MRCI, and ADC(3) levels of theory in connection with the aug-cc-pVDZ basis set using an underlying Mulliken-style population analysis.

State	CASSCF Q_a^t	MRCISD Q_a^t	ADC(3) Q_a^t	CASSCF LOC_a	MRCISD LOC_a	ADC(3) LOC_a
$^3B_{2u}^+$ (^3L_a)	1.935[a]	-	4.593	3.133[a]	-	5.327
$^3B_{3u}^+$ (^3B_b)	1.127[a]	-	1.715	1.372[a]	-	2.235
$^1B_{3u}^-$ (^1L_b)	0.025	0.239	0.314	0.117	0.394	0.504
$^1B_{2u}^+$ (^1L_a)	0.712	0.559	2.198	1.279	2.424	4.133

[a] Computed using OpenMolcas.

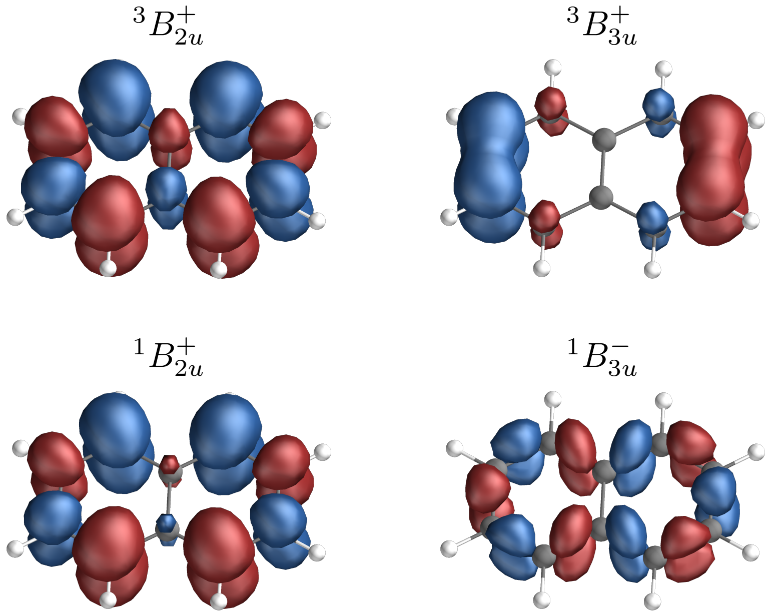

Figure S1: Transition densities of the lowest singlet and triplet excited states of naphthalene computed at the SA-CASSCF/aug-cc-pVDZ level using OpenMolcas.

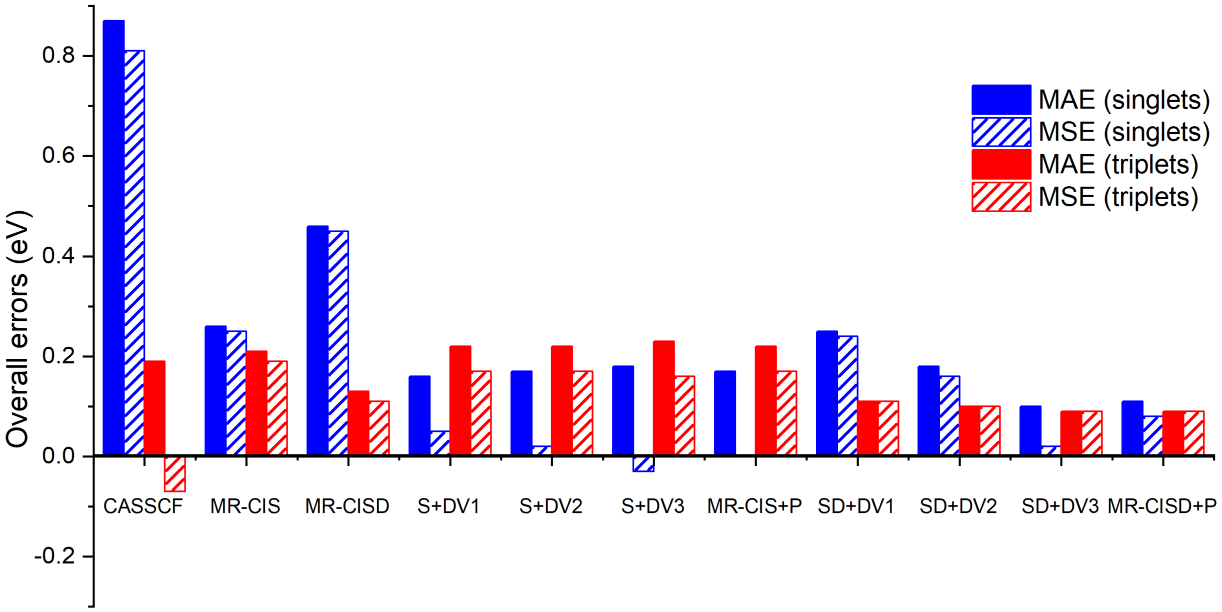

Figure S2: Overall errors of vertical excitation energies for CASSCF and various MR-CI variants (using the aug-cc-pVDZ basis set) computed for the eleven molecules considered in this work. Results are reported as mean absolute/signed errors (MAE, MSE) determined separately for singlet and triplet states.

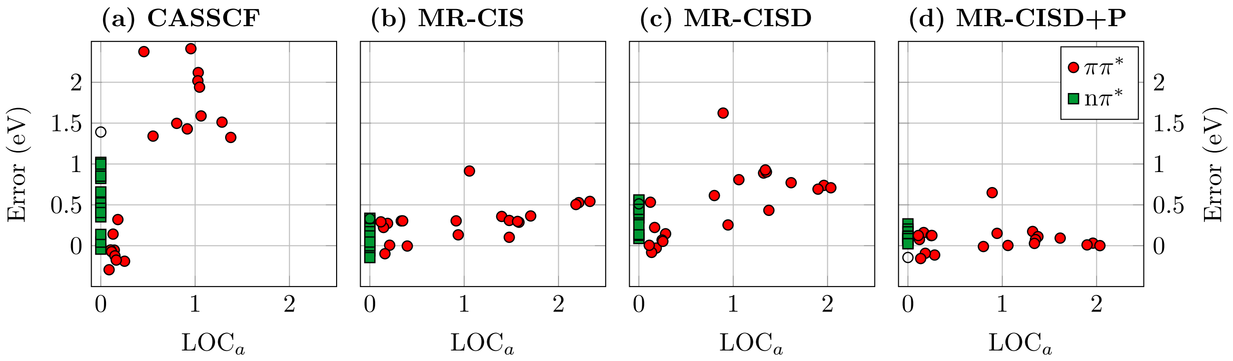

Figure S3: Errors of computed vertical singlet excitation energies plotted against the LOC$_a$ descriptor using (a) CASSCF, (b) MR-CIS, (c) MR-CISD, and (d) MR-CISD+P, all using the aug-cc-pVDZ basis set. States are grouped according to type: $\pi\pi^*$ (circles) and nπ^* (squares) states; the out-of-plane $\pi\pi^*$ state of cyanoformaldehyde is shown as empty circle.
